# Africa, an Emerging Exporter of Turmeric: Combating Fraud with Rapid Detection Systems

**DOI:** 10.3390/foods14091590

**Published:** 2025-04-30

**Authors:** Wilfred Angie Abia, Simon A. Haughey, Radhika Radhika, Brandy Perkwang Taty, Heidi Russell, Manus Carey, Britt Marianna Maestroni, Awanwee Petchkongkaew, Christopher T. Elliott, Paul N. Williams

**Affiliations:** 1 Institute for Global Food Security, The Queen’s University of Belfast, Belfast BT9 5DL, Northern Ireland, UK; w.abia@qub.ac.uk (W.A.A.); radhika01@qub.ac.uk (R.R.); hrussell14@qub.ac.uk (H.R.); m.p.carey@qub.ac.uk (M.C.); chris.elliott@qub.ac.uk (C.T.E.); p.williams@qub.ac.uk (P.N.W.); 2 Department of Biochemistry, Faculty of Science, University of Yaounde 1, Yaounde BP 812, Cameroon; tatybrandy13@gmail.com; 3 Co-Centre for Sustainable Food Systems, School of Biological Sciences, The Queen’s University of Belfast, Belfast BT9 5DL, Northern Ireland, UK; 4 Food Safety and Control Laboratory, Joint FAO/IAEA Centre of Nuclear Techniques in Food and Agriculture, Department of Nuclear Sciences and Applications, International Atomic Energy Agency, Wagramerstrasse 5, A-1400 Vienna, Austria; b.m.maestroni@iaea.org; 5 International Joint Research Centre on Food Security (IJC-FOODSEC), 113 Thailand Science Park, Phahonyothin Road, Khong Luang 12120, Pathum Thani, Thailand; awanwee@hotmail.com

**Keywords:** Africa, adulteration, *Turmeric*, exposure, fraud detection, portable X-ray fluorescence

## Abstract

Turmeric powder has gained widespread popularity due to its culinary and medicinal value and has become a target for economically motivated fraud. The history and exportation of turmeric in Africa were reviewed, and the safety issues of some toxic adulterants were discussed. Priority adulterants were determined from global food safety alerts. A systematic bibliographic search on Scopus, PubMed, Google Scholar, and Web of Science was performed to identify appropriate methods and techniques for authentication and safety. The quality of each study was assessed according to PRISMA guidelines/protocol. African turmeric exportation is on the rise due to recent insights into the suitability of local cultivars, soil and climate for growing high-quality turmeric, with curcumin levels >3%. There are limited data on turmeric adulteration for domestic consumption and export markets. This is important when considering that some turmeric adulterants may serve as risk factors for cancer following exposure. Global alert databases revealed lead chromate as the top hazard identified of all adulterants. Current techniques to detect adulterants are laboratory-based, and while efficient, there is a need for more rapid, field-friendly, non-destructive analytical tools for turmeric fraud/authenticity testing. This enables on-the-spot decision-making to inform rapid alerts. Portable technologies, such as portable X-ray fluorescence, were highlighted as showing potential as a Tier 1 screening tool within a “Food Fortress” systems approach for food safety, combined with validation from mass spectrometry-based Tier 2 testing.

## 1. Introduction

The herb and spice industry is under constant threat of economically motivated fraud due to the high economic values of the commodities it produces and the sector’s complex supply chains [1]. Colour, taste, anti-oxidant content, and aroma of spices, e.g., turmeric powder, are key characteristics that determine their quality/grade and influence the market price and consumers’ perception. Furthermore, aside from flavour, colour is responsible for the wide use of turmeric powder as a food ingredient in many food preparations [2] and influences customers’ decisions about brand quality when making purchases [3]. Thus, fraudsters illegally add similarly coloured chemical substances that may be toxic to humans to make the turmeric appear more attractive to consumers, along with masking additions of bulking agents; these processes are collectively termed economically motivated adulteration (EMA) [4]. This results in the turmeric being adulterated and potentially unfit for human consumption. The safety of turmeric is an important aspect to be considered alongside economically motivated fraud.

Turmeric (*Curcuma longa* L.), a botanical species from the *Zingiberaceae* family, has gained popularity in many parts of the world due to its extensive use in culinary practices, medicinal uses, and as a dietary supplement [5]. Historically, cultivation and use of turmeric has been centred in the Middle East and Asia [6]; lately, the African continent has also become an important exporter of turmeric. Of substantial concern is the increasing evidence that turmeric from leading producing areas is frequently adulterated (11% of imports to the EU) [7]. This is one of the factors that is creating emerging markets for export, especially when regulatory frameworks are less restrictive. Some of the adulterants identified, used for example as bulking agents, e.g., starch, are relatively non-toxic [8], whereas others are acutely toxic [9,10], such as lead chromate [11]. This systematic review reports on Africa’s emerging role as a turmeric exporter. Additionally, the review focuses on potential consumer exposure to adulterants and the associated health implications. It also collates global alert datasets for turmeric fraud and proposes suitable detection systems for the most frequently identified toxic adulterant.

## 2. Africa: An Emerging Exporter of Turmeric

A comprehensive literature review was conducted across multiple databases (Scopus, PubMed, Google Scholar, and Web of Science) to identify sources of information on turmeric in Africa. The review focused on the historical context, uses, varieties, processing methods, commercialization (importation and exportation), and adulteration of turmeric.

### 2.1. History, Use and Variety of Turmeric in Africa

**Figure 1:**
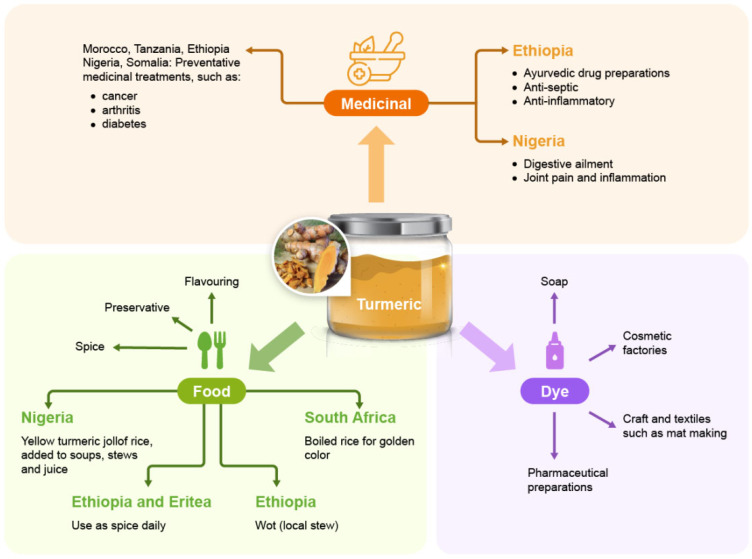
A range of the main uses of turmeric in Africa (Information sourced from [13,14,16,17].

During the 7th century, Arabs introduced turmeric to Morocco, where it was highly prized for cooking [12]. The spice first reached East Africa in the 8th century, initially in Madagascar via Borneo (South Asia). Subsequently, it was brought to Ethiopia via India in the 9th century [12]. By the 12th century, turmeric reached West Africa [13]. Historically, turmeric has been used for medicinal and other purposes; e.g., in Egypt, turmeric was used for healing wounds and as a dye [12]. Generally, in Africa, turmeric is valued in food, medicine and as a dye (Figure 1). Due to Indian influence, turmeric made its way into Ethiopian cuisine, where it was used in sauces such as “wot”, a local stew [13,14,15]. Nigerian immigrants in South Africa from the diaspora are known to consume and sell herbal concoctions of the rhizome bottled in alcohol for different ailments such as joint pain and inflammation [12]. Also, within Nigeria, it is becoming increasingly popular to blend spices with fruits to form a juice mix, and this practice is likely to spread across the West African sub-region [16].

An important feature of turmeric is the content of curcuminoids: these are a class of plant secondary metabolites that include curcumin, demethoxycurcumin, and bisdemethoxycurcumin, all isolated from turmeric. Many researchers have shown that curcuminoids have various biological activities, such as anti-oxidant, anti-cancer, anti-arthritis, and anti-inflammatory activities. There are several cultivars or varieties of *Curcuma longa* cultivated around the world. The curcumin content of turmeric is the most important factor in determining the development and cultivation of turmeric, and it has an important effect on the final price of turmeric.

In Ethiopia, a local variety of turmeric is used, and it has a curcumin content of 4% [18]. The Tepi National Spices Research Centre in Ethiopia introduced the ‘Dame’ variety in 2007 and the ‘Bonga 51/71’ and ‘HT3/2002’ varieties in 2018 [19]. The curcuminoid contents of each variety were as follows: Bonga 51/71 (6.49% m/m), HT3/2002 (5.12% m/m), and Dame (6.81% m/m) [19]. The two main varieties used in the world market are Alleppey and Madras, which differ from the varieties used in Ethiopia [20]. Alleppey is predominantly imported by the United States, where it is mostly used as a spice and food colorant. It contains 3.5–5.5% volatile oils and 4.0–7.0% curcumin [20], whereas Madras turmeric contains 2% volatile oils and 2% curcumin. This type of turmeric is preferred in the British and Middle Eastern markets as its more intense, brighter, and lighter yellow colour makes it more suitable for mustard paste and curry powder or paste used for meals [20]. These high curcumin levels and novel brands in the world turmeric space suggest Africa has the potential to be a major actor for turmeric production and exportation.

### 2.2. Turmeric Supply Chain (Top Exporters and Importers) in Africa

**Figure 2:**
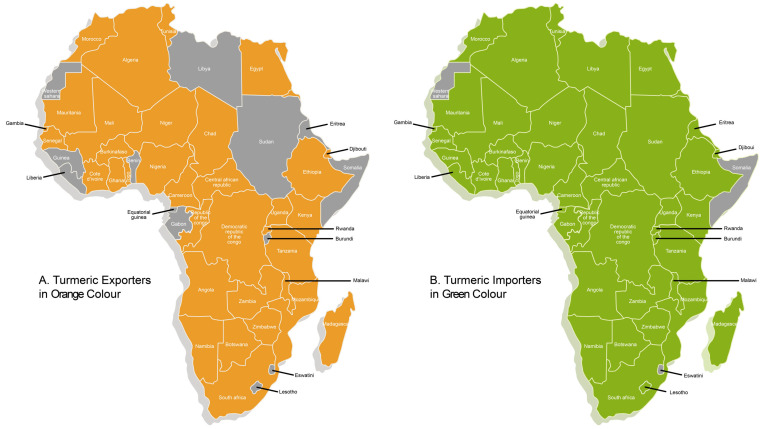
African Map showing Nations identified as Turmeric Exporters (**A**) or Importers (**B**) in 2022 and 2023 (more data on Africa’s top exporters and importers are available in Table A1 and Table A2, respectively, with percentage contributions; Source: designed based on the Observatory of Economic Complexity (OEC) [21]).

**Figure 3:**
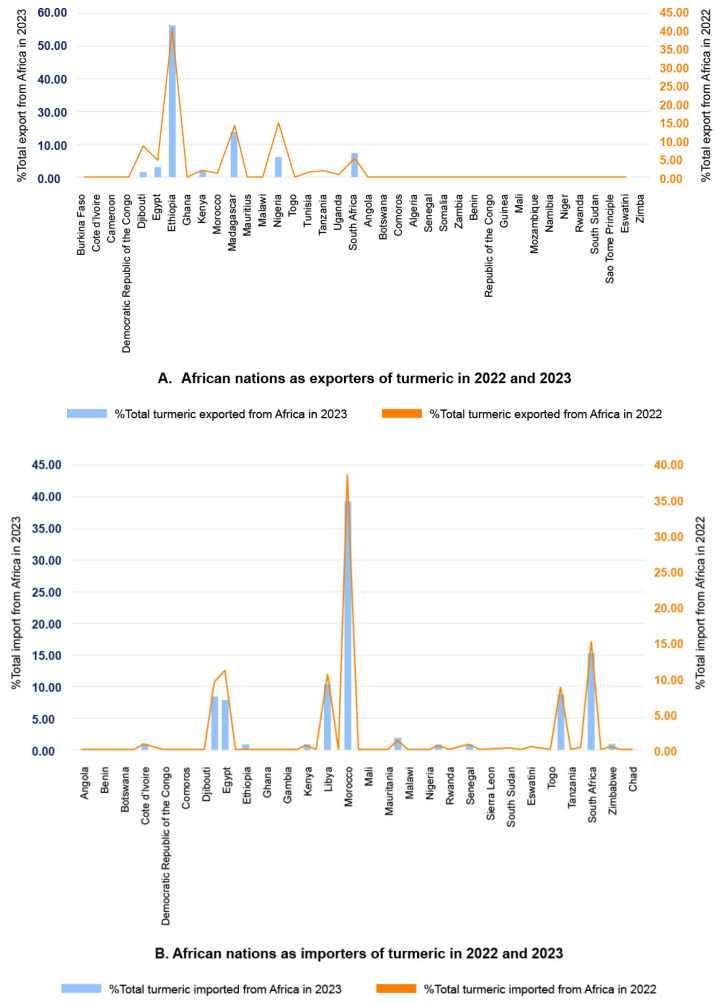

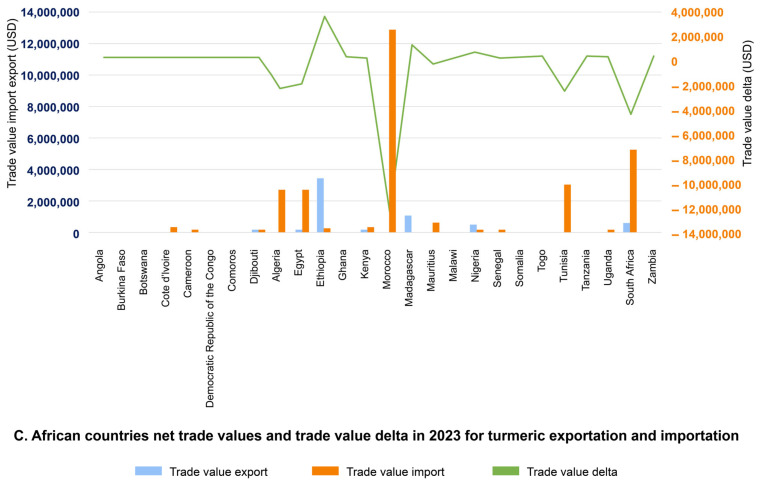
(**A**–**C**). Turmeric Trade Statistics in 2022 and 2023 (**A**). African Nations that were Exporters of Turmeric in 2022 and 2023; (**B**). African Nations that were Importers of Turmeric in 2022 and 2023; (**C**). Net Trade Values and Trade Value Delta of African Nations that were Exporters and Importers of Turmeric in 2023; Source: [21]).

In Africa, ~70% (38/54) of the countries exported turmeric in 2022 or 2023 (Figure 2A), with Ethiopia, Madagascar, Nigeria, South Africa, and Djibouti as the top five exporters over this period (Figure 3A) [17,21] (Table A1). These countries export to many different countries around the world (Table 1). Likewise, almost every country in Africa imported turmeric in 2022 or 2023 (Figure 2B), with Morocco, South Africa, Libya, Egypt, and Ivory Coast as the top five importers (Figure 3B) [17,21] (Table A2). The examination of export and import figures of turmeric is a useful tool within the context of food fraud as it may provide indications of fraud. For example, if more turmeric is being sold than is produced, this is a clear indication that a deeper investigation is needed and potentially some kind of food fraud is occurring. Between 2017 and 2021, African turmeric exports had an average growth rate of 18% per year [17]. This highlights a significant growth to date and emphasizes future potential in the market, which may indicate that a positive trade value delta (exports–imports) in the years ahead, relative to the net trade in 2022 and 2023, could reasonably be expected in some exporting countries (Figure 3C).

**Figure 4:**
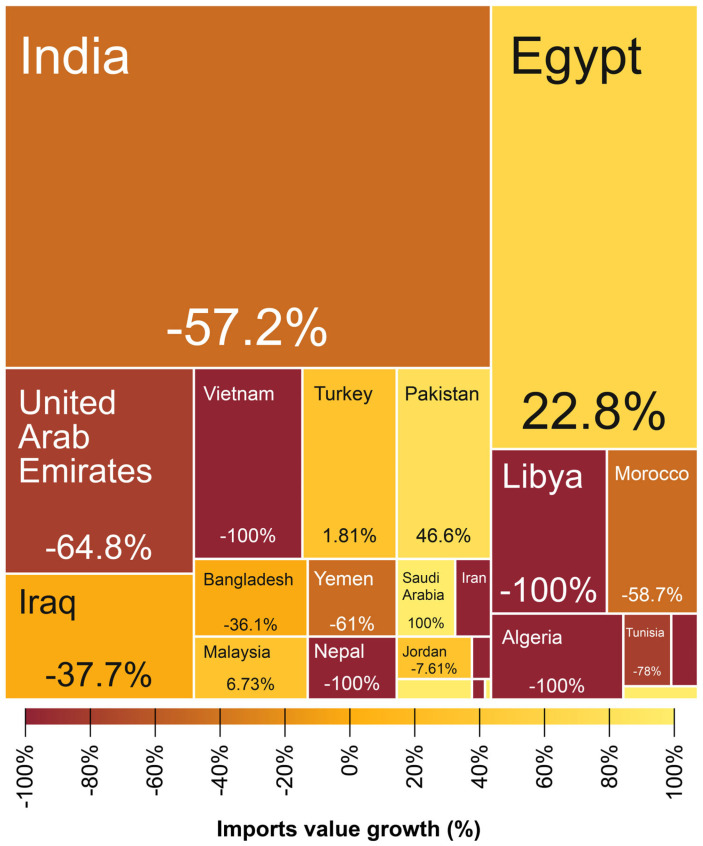
Percentage growth and degrowth of countries importing Ethiopian turmeric from 2021 to 2022 [21].

In Ethiopia, farmers only began systematic production of turmeric on a commercial scale in the 1970s [12]. Before 1972, turmeric was imported into this country [12]. Since 2014, turmeric production and productivity increased in Ethiopia in the southwestern part of the country (Sheka, Benchmaji, and Keffa zones) due to the bacterial wilt disease on ginger, resulting in farmers shifting to turmeric production [14,28]. Although productivity is lower compared to India [17], the Tepi National Spices Research Centre in Ethiopia revealed that the country’s turmeric production and processing methods provide a very high-quality product based on curcumin levels, and the resulting turmeric is of excellent quality [14]. This may be justified by the increasing number of countries importing turmeric from Ethiopia, including the World’s major turmeric exporter, India, despite fluctuation in import value growth (Figure 4). While in southwestern Ethiopia, turmeric is mainly produced by smallholder subsistence farmers, making up a significant proportion of their income [14], the cultivation of turmeric for commercial purposes is growing and is likely to become a cash crop elsewhere in Ethiopia [14].

It is predicted that the global market of curcumin, the active component of turmeric, will increase by 16.1% annually between 2021 and 2028 [17,29]. Within Africa, Nigeria and Cameroon are potentially going to increase their turmeric production and, consequently, their exports due to favourable soil and climatic conditions [12,16,17]. The Nigerian government is promoting the production of turmeric [30]. However, it has been reported that there is a lack of knowledge of cultivation technology among farmers in Nigeria [12], and this may hinder production levels.

### 2.3. Processing of Turmeric in Africa

**Figure 5:**
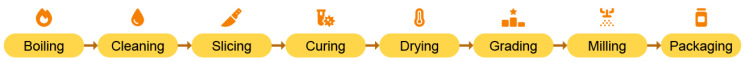
Typical turmeric processing after harvest (information sourced from [16]).

Turmeric undergoes various processes that can vary depending on the country, after harvest, and before it can be used (Figure 5). In Ethiopia, harvest times are in December and January [14].

Traditionally in Ethiopia, after harvest, turmeric rhizomes are put in a bag and beaten on a hard surface, or in an improved process, a hand-operated barrel or polishing drum is used [14] to straighten the rhizomes and eliminate scales and root bits. In Ethiopia, farmers set aside some rhizomes to grind them uncooked using a mortar and pestle to produce turmeric juice, which is mixed with lemon juice [14]. This yellow solution is then applied to boiled rhizomes to increase the colour of turmeric and to provide natural protection to the rhizomes from a potential weevil attack, thereby increasing storage time [14]. Adding the natural protective solution is a mechanism for farmers to add value to their products. However, large/international traders are less interested in purchasing coated turmeric rhizomes; hence, it is no longer practiced widely by farmers [14]. The larger traders complete processing themselves in their warehouses after purchasing dry rhizomes from farmers to protect and ensure product quality [14]. In Bangladesh, Forsyth et al. [9] reported that farmers can increase their profits by asking the polishers to add yellow pigments. It is well known that adulterating turmeric with these yellow pigments (such as lead chromate, metanil yellow and Sudan dyes) can be harmful to consumers [9,31].

### 2.4. Turmeric Adulteration Affecting African Exporters

The literature on the adulteration of turmeric with potential substances such as metanil yellow and lead chromate in the larger exporters of turmeric was not found. However, respondents in a study conducted in Ghana reported hearing metanil yellow was being used to adulterate turmeric powder [32]. In the study, respondents were asked about common adulterants used in food items. Four respondents mentioned turmeric was adulterated, two reported colour, one reported sawdust, and another reported a yellow dye as the adulterant being used. A study conducted in East African countries (Ethiopia, Kenya, and Uganda) using portable X-ray fluorescence (pXRF) reported lead content <1 mg/kg, with the authors suggesting adulteration with pigments was non-existent [33]. However, lead/lead chromate was the only adulterant considered in this study. Contrastingly, another study conducted in Ethiopia using inductively coupled plasma-optical emission spectroscopy (ICP-OES) reported levels of lead (16.1 ± 0.500 mg/kg) in turmeric, which is well above the maximum permissible limits set by the European Commission for root and rhizome spices (1.5 mg/kg) [34]. However, this could be due to environmental contamination through water and/or soil rather than turmeric adulteration with lead chromate. Lead has been found in high amounts in soil in Ethiopia, contributing to high levels present in fruits and vegetables, both exceeding the FAO/WHO maximum permissible limit [35,36].

A major challenge of turmeric exportation from Africa vis-à-vis lead is that regulations for turmeric and spices vary across different regions and organizations. The US FDA has set a maximum limit of 10 mg/kg for lead in naturally sourced food colours, including turmeric [37]; no national standard exists for lead in spices. The European Commission established a limit of 1.5 mg/kg for lead in root and rhizomes spices [34]. The FAO/WHO Codex Alimentarius Commission (CAC) has set a maximum level of 2.0 mg/kg for lead in spices [38]. The African Union is developing food safety guidelines, but specific lead limits for spices are not yet available [39]. For individual African nations, information on lead limits in spices is limited. South Africa follows Codex standards for lead in spices [39]. Specific regulations for Cameroon, Ethiopia, Nigeria, and Morocco regarding lead limits in turmeric or spices were not found in any search results from the literature. For example, Cameroon has a Framework Law on Food Safety, which was passed in 2018 [40]. However, there is no regulation for lead in turmeric or spices. These nations generally follow maximum limits set by trade partners; e.g., Cameroon follows the European Union, which has set its own regulatory limits.

The literature on turmeric adulteration in the top and upcoming exporters of turmeric in Africa (Ethiopia, Djibouti, Madagascar, South Africa, Nigeria, and Cameroon) was not found. However, it is known that adulterants, such as metanil yellow and lead chromate, are being detected in turmeric from other origins, e.g., Bangladesh and India. It is therefore important to ensure that emerging exporters may be trading only authentic and quality turmeric, and portable, field-friendly analytical methods should potentially be developed to ensure food safety. While it is not a food safety issue, the quality of traded turmeric powder should also take into account the curcuminoid content and, therefore, promote turmeric powders made from *Curcuma Longa* species only.

## 3. Turmeric Adulterants: Focus on Exposure and Associated Health Implications

### 3.1. Exposures to Adulterants in Turmeric and Associated Health Implications

**Figure 6:**
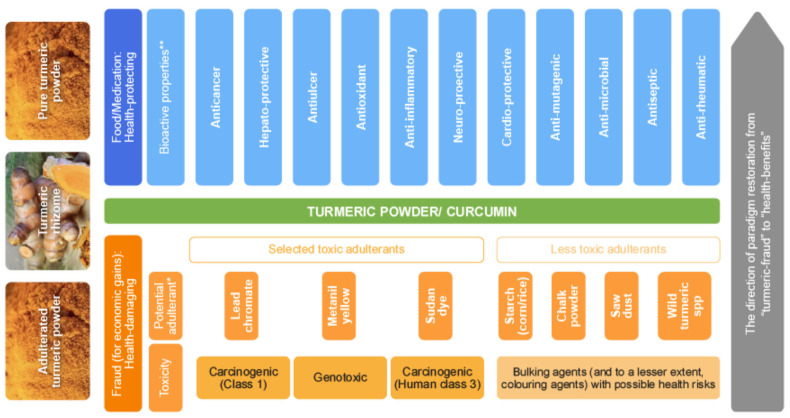
The good and bad of turmeric powder: An overview of the turmeric (*Curcumin longa*) shift (from authentic turmeric powder, i.e., “turmeric-for-food-and-health” to not-authentic turmeric powder, i.e., “turmeric-fraud-harm-to-consumers-health”) paradigm. Carcinogenic (Class 1): [11]; Genotoxic: [41,42]; Carcinogenic (Class 3): [43]; Bulking agents with possible health risks: [8]. Bioactive Properties **: As reviewed in detail by Xu et al. [44], Yeung et al. [45], and Sharifi-Rad et al. [46]. Potential Adulterants *: A detailed list with references is presented in Table A1. For example, lead chromate by Lopez et al. [47] and Forsyth et al. [10]; Metanil yellow by Nath et al. [42] and Kumar et al. [48]; and Sudan dye reported by Di Anibal, Ruisánchez and Callao [49] and Ullah et al. [50]. Note: The maximum permissible limit (ML) for Metanil yellow in food is 100 mg/kg [51], while the ML of Sudan dye I-IV in food is 0.5 mg/kg [52].

Studies focused on exposure to adulterants via turmeric intake and health implications were thoroughly searched across various search engines (Scopus, PubMed, Google Scholar, and Web of Science). The associated health implications range from mild to severe depending on the type of adulterant, exposure duration, dose intake, and consumers’ health status. Figure 6 provides a holistic summary of the benefits of authentic turmeric and the health effects following dietary exposure to various adulterants. Compared with other adulterants, lead chromate exposure has gained wide attention as a public health issue. While exposure assessments are crucial in understanding the potential risks, techniques for field detection of the presence of adulterants in turmeric are less developed.

### 3.2. Exposures to Lead (Through Lead Chromate) Adulterated Turmeric and Associated Health Effects

The use of lead chromate to enhance the yellow colour appearance of turmeric is increasingly being reported. However, lead can be present in the soil where turmeric rhizomes are growing.

One study suggests that the uptake of lead from the soil into the turmeric is a possible but unlikely source of contamination, as previous studies estimate the maximum uptake of lead into the root of the plant to be approximately 10% [53]. According to Forsyth et al. [54], lead to chromium molar ratio of approximately 1:1 in turmeric may suggest lead chromate adulteration. This scenario was found in turmeric adulterated with lead chromate across South Asia [10].

The consumption of lead in spices, e.g., turmeric, is a public health concern worldwide when considering the associated negative health effects [55]. In general, such adulterations only take place when turmeric rhizomes are transformed into powders. It is, in fact, well known that the addition of bulking agents and colourants is only applicable to homogenized turmeric. According to available datasets, lead chromate is the primary adulterant in turmeric powder partly due to its low price and non-specific regulatory/maximum limit (ML) in turmeric powder [9]. In the European Union (EU), the ML of lead in root and tuber vegetables (0.1 mg/kg in fresh turmeric rhizomes) and dried root and rhizome spices (1.5 mg/kg) has been recently established by the EU and expressed in the Commission Regulation (EU) 2021/1317 of 9 August 2021 amending Regulation (EC) No 1881/2006 as regards maximum levels of lead in certain foodstuffs [34]. It is worth noting that chromium is also particularly toxic in its hexavalent state, e.g., it may serve as a risk factor for lung cancer [56,57,58]. In addition, hexavalent chromium was reported in turmeric in one study in India [47]. However, the primary concern for lead chromate in this review is due to its much greater toxicity.

Exposure to lead from turmeric powder adulterated with lead chromate has been shown to cause neurological abnormalities, developmental delays, and cognitive impairments among exposed individuals [49]. In southern Asian countries, lead has been recognized as a risk factor for preterm birth due to increased consumption of turmeric powder by pregnant women, aiming at improving their nutrition and health [54,59]. Furthermore, prolonged exposure to lead may exert damage to the bones, brain development, and reproductive and respiratory organs [60,61,62]. Exposures to low levels of lead (<3.5 µg/dL) have been associated with deterioration in cognitive ability and behaviour change [63] as well as poor performances in school and reading challenges, amongst others [64,65,66,67]. There is no safe blood lead level in children [68,69], and unfortunately, anthropogenic lead exposure (Table 1), partly through intake of lead-contaminated turmeric powder has been indicated to be the primary factor contributing to elevated lead levels among children in Bangladesh (N = 309; aged 20–40 months; blood lead levels > 5 µg/dL) [54,59,70], India (an est. 275 million children, aged 0–9 years, blood lead levels ≥ 5 μg/dL) [71], and the US [72]. For the US, blood lead levels of 2.9–12.7 µg/dL in five children in Kansas City were associated with exposure through intake of lead (6.86 μg/g, Table 1) adulterated turmeric powder [73]. Likewise, in Las Vegas in 2019, it was speculated that childhood lead poisoning might be associated with the consumption of turmeric contaminated with lead [74]. When considering that blood lead in a gestational exposure may cross the placenta to the fetus [75,76,77], pregnant women directly place their unborn babies in danger of lead exposure, although disclaimed by a recent review [78] that calls for more evidence in this direction.

The adulteration of turmeric with lead chromate raises concerns not just for regions involved in its production and consumption but also for those linked to its importation [79]. For example, turmeric powder bought from India (median: 0.71, max: 6504 mg/kg) revealed more than three times higher median lead levels when compared with those bought from the US (median: 0.19 mg/kg) [80]. Additionally, the consequences of toxic metals, particularly lead chromate adulteration, extend beyond endangering public health as it also impacts the integrity of global food supplies and companies that unwittingly become involved in supply chains that sell such products [81]. This underlines the necessity of improving food safety and preventing fraud crimes, including strategies to reward farmers and implementing stringent monitoring initiatives along with more robust regulation enforcement. Going forward, there is a need for more studies to investigate the source of lead contamination in spices [82] in general and turmeric powder in particular. Although this requires highly sophisticated techniques to perform such analysis, simple “direct-scan” methods, linked to chemometrics models established with real authentic samples, may be speculated as the technique of choice for rapid detection of adulterants, especially for lead, on the field, thus, eliminating the chances of adulterated turmeric spreading in local markets, and/or crossing trade borders.

## 4. Detection of Adulterants in Turmeric Powder

### 4.1. Online Systematic Searching, PRISMA Analysis

A systematic approach was used to search existing documents to identify articles that reported on turmeric powder (branded or open source) adulteration fraud, adulterant detection methods, and associated consumers’ exposure health implications. The Scopus, PubMed, Google Scholar, and Web of Science search engines were used to source relevant articles written in the English Language with no date limits (i.e., from database inception to 7 February 2024). During the process, specific keywords and the combination of these keywords that appeared either on the title and or abstract (“turmeric” AND “adulteration” OR “detection”) were screened with the aid of Boolean operators and included. The search results were each imported to a Covidence (reference managing database). The “Preferred Reporting Items for Systematic Reviews and Meta-Analyses” (PRISMA) [83] guidelines/protocol were applied to transparently and consistently present the methods and results of the studies included in this review. All authors participated in the development of the inclusion and exclusion criteria.

The full text of each relevant study was considered for review after screening their titles and abstracts. Inclusion criteria included turmeric, adulteration, and detection. Owing to the limited number of articles on turmeric powder adulteration and detection of adulterants, inclusion criteria were broadened to include articles that only presented data on turmeric adulteration. Exclusion in the initial screening included, but were not limited to, articles that focused on curcuminoid contents, considered turmeric as an adulterant in other spices, compared turmeric species and or products, medicinal usages of turmeric, and those not focused on turmeric or written in another language (e.g., Spanish, French, Danish, Italian, German, and Japanese) other than English language. Full-text review exclusions included articles with no data and only expert opinion, documented reviews on turmeric, conference paper compilations, etc.

**Figure 7:**
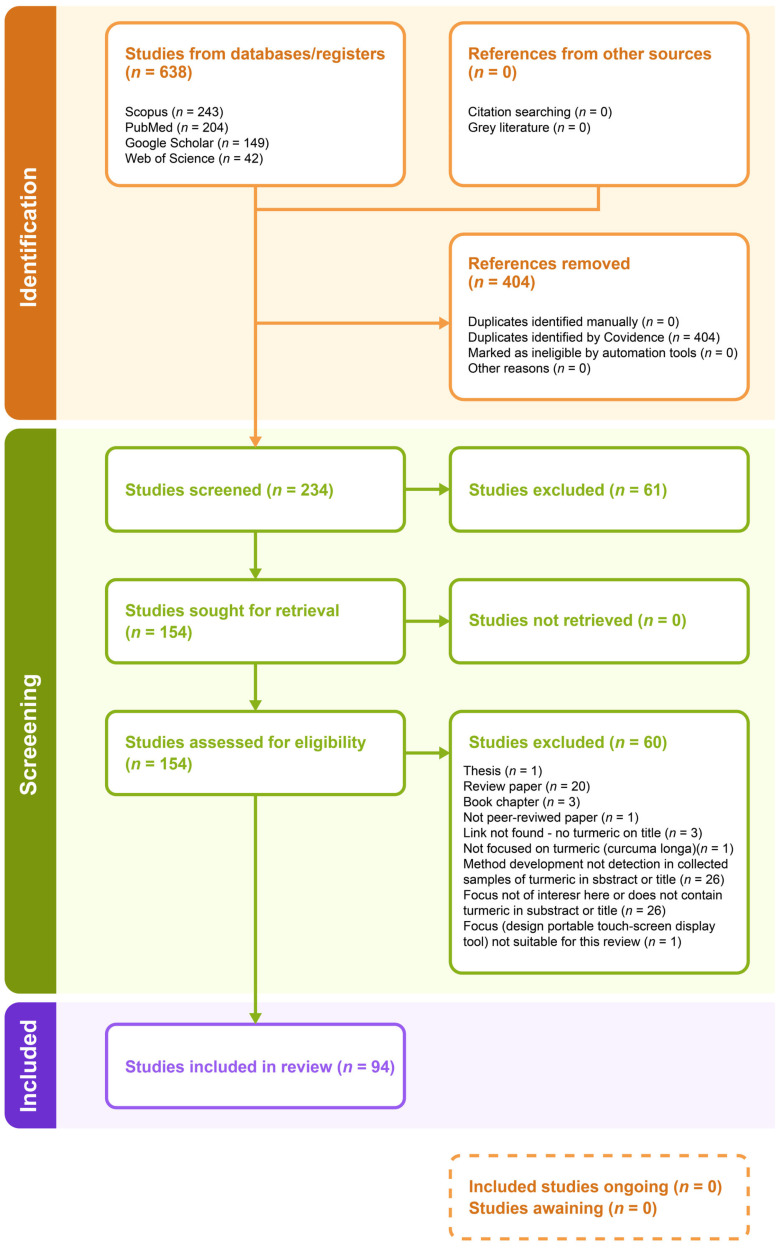
PRISMA flow diagram.

Based on the systematic literature search, 638 article references were identified and imported into Covidence software, where duplicates were removed (*n* = 404) during the PRISMA identification process. The full texts of the remaining 234 studies were submitted for the screening process (61 studies were excluded, and 154 were retrieved). The retrieved studies were assessed for eligibility, from which 94 eligible studies were included in the review, and 60 were excluded (Figure 7).

### 4.2. Turmeric Adulteration Detection Methods

Generally, turmeric powder adulterants reported in the literature include starch (from diversified tubers such as cassava and cereal-based foods, e.g., corn), diversified types and classes of *Curcuma* powder (including spent turmeric, synthetic turmeric, and *Curcuma* spp., e.g., *C. zedoria* and *C. mangga*) that closely mimic *Curcuma longa* L. powder, yellow-mimicking substances (such as Yellowstone powder, and sawdust), and the azo dyes (including metanil yellow, Sudan dye, aniline dye), and lead chromate (Table A1). The methods (either single or in combination) used to detect these adulterants in turmeric powder include physical and chemical methods of the Food Safety and Standards Authority of India (FSSAI), microscopy, molecular, chromatographic (gel permeation chromatography, GPC, and thin-layer chromatography, TLC), spectrometry (e.g., ICP-MS), and spectroscopy (such as Raman, laser-induced breakdown (LIB), near-infrared (NIR), Fourier-transform infrared (FT-IR), X-ray fluorescence (XRF), etc.). In some cases, the method used was in combination with multivariate analysis tools (specifically the principal component analysis PCA and the partial least squares discriminant analysis PLS-DA). Some of these techniques employed in the detection of adulterants and authentication of turmeric have been reviewed by Sasikumar [84]. Spectroscopy is amongst the most frequently used approaches for detecting fraud and authentication of turmeric powder. It has the advantage of being a non-destructive field-friendly analytical technique and thus provides an option for rapid field decisions.

Generally, an overview of applications, performance criteria, and the challenges of various physical, chemical, and molecular techniques used for adulterant authentication in food, spices, and herbs have been described in detail [85,86,87,88]. Some of the methods include but are not limited to, LIB spectroscopy (LIBS) [89], Raman spectroscopy [90], X-ray powder diffraction (XRD) [91], Fourier-transform infrared (FT-IR) spectroscopy coupled to chemometric analysis [8], and the flame atomic absorption spectrometry (FAAS) [92]. In addition, some of these techniques, such as UV-visible spectroscopy, developed as a simple and rapid detection tool, have inherent limitations, such as measurement errors due to high matrix interferences and poor limits of detection [93,94]. Likewise, FT-IR and NIR exhibit similarly high limits of detection to UV-vis spectroscopy, which exceeds the regulatory threshold with poor sensitivity for detecting lead adulteration in turmeric and a lack of validation capabilities for quantitative analysis of toxic metals [79,95]. On the other hand, LIBS [48] and XRD [91] have been trialled recently for Pb detection in spices. However, thus far, it has only been validated for screening purposes.

Furthermore, the reliability and usefulness of the information generated by the aforementioned analytical techniques increase when exploited in combination with other non-destructive techniques used to detect illegal adulteration of spices with various harmful adulterants. Therefore, considering this, the application, characteristics, advantages, limitations, and development of multi-source and non-destructive analytical techniques such as vibrational spectroscopy (including FT-IR, FT-MIR, near-infrared NIR, Raman, and hyperspectral imaging HSI), and the electronic sensor technology (e.g., electronic nose and electronic tongue), amongst others e.g., multivariate analysis, to assess the quality and authenticity of spices have been reviewed extensively [1,88,96]. Furthermore, the purpose of each step in the process of authentication, including the use of non-destructive analytical techniques (to measure chemical information in samples), data processing (to remove artifacts and improve the accuracy of results), data fusion (for multi-sourced data to further improve the accuracy of results), and building of models (for exploration, prediction or classification of samples as authentic or suspicious) have been highlighted [88,96]. The application of these methods, which are mostly laboratory-based, is to provide reliable measurements for rapid decisions on whether the tested turmeric is adulterated or not. However, rapid detection systems for field testing by food industries and food safety agencies are ideal for reducing exposure to toxic adulterants. Considering the complexity of developing such techniques, the emphasis for method development should be on the most frequently reported adulterants.

### 4.3. Frequently Reported Turmeric Adulterants in Rapid Global Alert Reports

**Figure 8:**
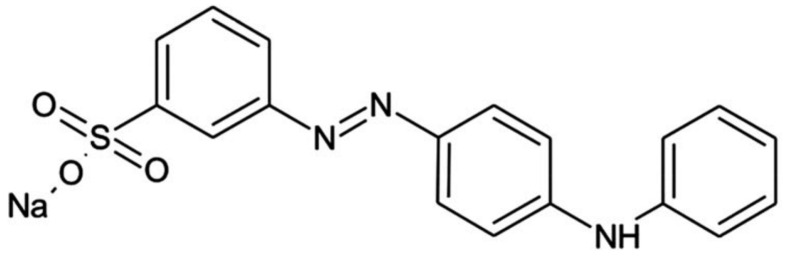
Structure of metanil yellow (Adapted from [31]).

According to the economically motivated adulteration (EMA) Hazard Identification Reports (HIR) (Source: Decernis Food Fraud Database) and the Rapid Alert System for Food and Feed (RASFF), the most frequently suspected toxic chemical adulterants in the alerts were metanil yellow and lead chromate (Table 2). Metanil yellow (Figure 8), which featured in 39.74% of the total alerts, is a bright yellow synthetic toxic azo dye and is sometimes added to turmeric powder, which poses potential health hazards such as cancer and damage to the gastrointestinal tract and liver [31]. The International Agency for Research on Cancer and FAO/WHO classifies metanil yellow as a class II potent carcinogen [97,98]. Some health effects caused by the consumption of metanil yellow include tumours, neurotoxicity, hepatocellular carcinoma, lymphocytic leukaemia, and other chronic ailments [98].

Lead chromate and lead are featured in 59% of the total alerts (Table 2). Additionally, considering the HM Global Alerts from 2010 to the present, lead is the most frequent (*n* = 165 alerts) toxic metal suspected in spices, and turmeric alone had 58 alert cases, followed by curry powder 29 alerts and cinnamon 28 alerts (FoodAkai platform). Therefore, it is of great importance to examine suitable approaches to detect fraud involving lead chromate in turmeric.

### 4.4. Suitable Detection Systems for Lead in Turmeric Powder

Turmeric powder adulteration with lead chromate is increasingly being assessed using costly, time-consuming and non-field-friendly techniques (Table A3), which may not significantly contribute to rapid field decisions. Therefore, potentially non-destructive analytical tools for rapid field decision-making are of high interest in reducing potential exposures to adulterants. In this study, selected via PRISMA methodology, the focus was on the rapid detection and screening of lead in adulterated turmeric powder using portable X-ray fluorescence (XRF) analyzers. The focus was also on the confirmatory approach with inductively coupled plasma-mass spectrometry (ICP-MS) (or inductively coupled plasma-optical emission spectroscopy, ICP-OES), which was not given much attention in previous reviews on food and spices [85,86,87,88]. Table 3 summarizes the various reports on lead adulteration in turmeric powder examined using ICP-MS and/or XRF.

The ICP-MS, and, in particular, XRF spectroscopy, have been exploited in laboratory settings for many years in investigating levels of multi-elements in several domains, including geological, forensic, and archaeological sciences [99,100]. Additionally, the strength and limitations of portable XRF for the quantitation of elemental composition of archeological sediments and ceramics [99], as well as its applications in applied geochemistry [101] and consumer products [102], calibration for elemental analysis in fertilizers [103], food [104], feed and agricultural analysis [105] have been extensively reviewed. Moreover, the impact of sample preparation methods for geochemical analysis of soil and sediments [106,107] and matrix effect correction [108] using handheld XRF have been reported. Palmer [102] has extensively discussed the potentials of ICP-MS and pXRF as techniques of choice for multiple toxic and nutrient elemental analyses.

Generally, within the spices industry and amongst regulatory bodies, validated and accredited mass spectrometry, particularly ICP-MS, is widely used for multi-elemental analysis due to its specificity and sensitivity with high sample throughput [109]. ICP-MS, which has been used to measure lead chromate levels in turmeric [9,47,54,72,79,110], is not only time-consuming, but it involves elaborate sample preparation steps and requires skilled laboratory staff [111,112]. In addition, its substantial purchase/installation costs, coupled with the high resource requirements (i.e., Ar gas, electricity, high purity reagents) of ICP-MS, may pose a challenge for use as a routine analysis approach for many low-income countries, which include many of the primary producers of turmeric [82]. Considering its relative effectiveness, it is suitable as a reference instrument for the confirmation of the presence and level of toxic lead in any suspicious turmeric samples.

On the other hand, XRF is gaining wide recognition as the most practical solution for detecting toxic metal adulteration in spices [113]. Among XRF techniques, the energy dispersive pXRF has gained interest due to its portability, allowing for in situ analysis in the field, and improved detection limits between 1 and 8 mg/kg Pb, which are close to the regulatory limits (between 1 and 10 mg/kg Pb) set by various international bodies including India and the EU [47,114,115]. Furthermore, the pXRF is relatively cost and time-beneficial for screening and does not necessarily require a skilled laboratory staff to operate. Forsyth et al. [9] and Lopez et al. [47] have demonstrated that handheld XRF analysis provides information that can enhance field detection of lead in turmeric powder and thus may contribute to a reduction in lead exposure amongst various consumer populations. However, there is a need to explore the functionality of the pXRF to improve calibration to detect lead levels below the ML of 1.5 mg/kg [34], thereby rendering it suitable for rapid screening analysis and, thus, improving the decision-making approach in the spices industry.

**Table 3:** Lead contents in speculated lead chromate adulterated turmeric powder sourced from various geographic areas and measured using ICP-MS (OES) and XRF.

Country (Sample Origin)	Method of Detection	Adul Terant	Frequency (%)	Mean Level mg/kg	Range or Max, mg/kg	References
Bangladesh	XRF	Pb ^ⱡ^	/	80	<LOD-483	[70]
US (purchased from Bangladesh, India, Nepal, Pakistan, Morocco, US)	ICP-MS	Pb	76/105 (72)	160	2700	[116]
Bangladesh (9 districts)	ICP-MS, XRF	Pb ^ⱡ^	16/140 (11)	1152	/	[9]
Bangladesh	ICP-MS, XRF	Pb ^ⱡ^	52/200 (26)	690	/	
India	ICP-MS, XRF	Pb ^ⱡ^		0.2	0.7	
Republic of Korea	ICP-MS	Pb	13 (100)	0.09	0.04–0.97	[114]
Republic of Georgia	ICP-MS, pXRF	Pb	2 **	1897.06 *	466–3328	[82]
India—Penta, Bihar	pXRF	Pb	128 **	114.6	<44–>363	[71]
Bangladesh, Pakistan	ICP-MS, pXRF	Pb ^ⱡ^	11 **	857	1.04–4221	[47]
India	ICP-MS, pXRF	Pb ^ⱡ^	11 **	1118	0–5279	
US (Kansas)	ICP-MS	Pb	1 (100)	6.86	6.86	[73]
US (also purchased from India)	ICP-OES	Pb	71 **	205.33 ^Ⱡ^	0.06 ^Ⱡ^–6504	[80]
	ICP-OES/-MS	Pb	13 **	0.12	0.07–0.28	
South Asia (India, Pakistan, Sri Lanka, and Nepal)	ICP-OES/-MSpXRF	Pb	51/356 (14)	3 * (>LOD: 2)	2936	[10]

*: Median; **: Number of observations; ^Ⱡ^: Multiyear sampling 2011–2020; ^ⱡ^ Pb: Lead value estimated from lead chromate adulteration based on a 1:1 molar ratio of lead to chromium in the samples; ICP-MS: inductively coupled plasma-mass spectrometry; ICP-OES: inductively coupled plasma-optical emission spectrometry; pXRF: portable X-ray fluorescence. Note: ICP-MS is a laboratory-based instrument, and pXRF is both a laboratory-based and portable (or handheld) or field-based instrument.

The different setup of instruments, such as energy-dispersive XRF (ED-XRF), is of utmost importance in the field of elemental analysis as additional parameters, such as vacuum or filters, might impact the quality (sensitivity) of the acquired data. Some instruments operate under vacuum conditions to enhance sensitivity, while others analyze samples in ambient environments, which requires careful assessment of atmospheric interferences [117]. Filters used in the instruments help minimize undesirable interferences while improving the quality of the incident beam spectrum [118]. Elements present in low amounts in test samples result in a low signal-to-noise ratio, which limits the performance of portable XRF. Nonetheless, this limitation can be mitigated using secondary targets with increased analysis time [119]. Additionally, the choice between the fundamental parameter and empirical method for matrix correction impacts the accuracy of the results. While empirical methods are generally fast with high accuracy, their use is often limited by the concentration range of available reference materials [120]. The instrument geometry affects spatial resolution and representativeness. Therefore, the sample needs to be extremely homogenous for XRF analysis [121]. Each of these factors contributes to the overall quality of XRF data, along with proper calibration, matrix effects, and detector energy resolution. Where calibration materials are or closely resemble the samples to be screened, the empirical method via calibration curves would be more suitable [122]. Acquah et al. [103] have provided an extensive review of pXRF calibration, while Palmer [102] has extensively discussed legislative applications of XRF for elemental analysis in fertilizers and consumer products. Based on the above, proper lessons may be drawn to guide similar analysis on turmeric powder.

Altogether, in the hope that the screening (empirical) method of the pXRF can be further developed/calibrated to detect below the regulatory level of 1.5 mg/kg lead in turmeric powder as established by the EU, the pXRF is a potentially simple, rapid field-friendly non-destructive analytical tool for screening for elemental analysis, e.g., lead in turmeric. This is particularly important as it can be used globally, including research in infrastructural resource-limited and low–middle-income countries (LMIC), e.g., in Africa. Furthermore, should there be a need to confirm suspected samples, a validated and accredited ICP-MS method is recommended. Further details on the merits of pXRF and the ICP-MS have been well summarised by Palmer [102].

From the perspective of the “Food Fortress” system [123]—a framework designed to enhance food safety and traceability through advanced monitoring and testing protocols, and one that is practically applicable in large-scale food production and export operations where consistent quality control is critical—technology diffusion is feasible through government-backed policies, industry incentives, and partnerships with testing laboratories, especially in regions with emerging food industries aiming to meet international standards (e.g., Africa). In this context, portable technologies such as pXRF have shown potential as Tier 1 screening tools within a “Food Fortress” systems approach, complemented by validation through mass spectrometry-based Tier 2 testing.

## 5. Conclusions

Turmeric powder is well known as a golden spice that is used culinarily and medicinally. Its popularity has also made turmeric vulnerable to economically motivated adulteration. Reports on turmeric fraud from well-known turmeric-producing nations such as India in Asia are highly worrying, prompting alternative markets to be sought. Africa, in terms of supply, is a turmeric-underrepresented continent, revealing few but promising productions of turmeric worth exploring and encouraging as an emerging turmeric producer/exporter of more authentic and safer turmeric. This is particularly important when considering the potential health implications that may arise following dietary exposure, especially amongst children, to adulterated turmeric powder, e.g., lead chromate adulterated turmeric powder. Thus, as consumers’ demand for authentic turmeric powder continues to increase, spice industries and regulatory bodies need simple analytical tools that can be used in the field for rapid detection of adulterants such as lead chromate. Thus far, various techniques, including physicochemical, microscopy, chromatography, spectrophotometry and spectroscopy, have been employed to detect fraud. These techniques are mostly laboratory-based, implying that before analysis is conducted to confirm or suspect fraud, the turmeric might already be on the market. Although these methods have been very useful, there is a need for field-friendly detection methods that can be used globally, including in Africa. Various global alert reports revealed lead chromate as the most frequently suspected adulterant in turmeric. Based on that, the screening (empirical) method of the pXRF may be an ideal rapid, non-destructive, field-friendly analytical tool for the detection of lead (or lead chromate) adulteration in turmeric powder—in the hope that it can be further developed and calibrated to detect below the regulatory level of 1.5 mg/kg lead in turmeric powder. Additionally, there is a need to confirm that pXRF revealed non-compliant or suspicious turmeric to ensure that a validated and accredited ICP-MS method would be a reliable confirmatory tool.

## Figures and Tables

**Table 1:** Importing countries from the top African exporters and “upcoming exporters” in 2022.

Country Exporting	Importing Countries	References
Ethiopia	50% India, 8% Egypt, 6% Iraq, 5% Turkey, 5% Pakistan, 3% Malaysia, 2% Bangladesh, 1% Saudi Arabia, 1% Iran, <1% United States of America, <1% Kenya	[22]
Djibouti	88% India, 12% Malaysia	[23]
Madagascar	51% Germany, 49% France, <1% Italy, <1% Belgium, <1% Canada	[24]
South Africa	19% Zimbabwe, 12% Botswana, 8% Namibia, 7% Zambia, 4% Mozambique, 3% Australia, 1% Angola, 1% United Arab Emirates, <1% Ghana, <1% Congo, <1% Kenya	[25]
Nigeria	61% India, 33% United States of America, 6% Germany	[26]
Cameroon	100% France	[27]

**Table 2:** Occurrence frequencies of suspected adulterants in turmeric across the EMA HIR and RASFF between 2004 and 2024.

Adulterant	F in EMA HIR (*n* = 51)	F in RASFF (*n* = 27)	Total (%) (*n* = 78)
Lead chromate, lead	34	12	46 (58.97)
Metanil yellow	31	0	31 (39.74)
Starch (corn, rice, etc.)	18	0	18 (23.08)
Sudan dye (I-IV)	9	13	22 (28.21)
Other *Curcuma* spp.	6	0	6 (7.69)
Others (chalk, orange II, rhodamine, sawdust, etc.)	7	2	9 (11.54)

F: Occurrence frequency; n: Number of alerts.

## Data Availability

No new data were created or analyzed in this study. Data sharing is not applicable to this article.

## Abbreviations

The following abbreviations are used in this manuscript:

EMA	Economically Motivated Adulteration
FDA	Food and Drug Administration
OEC	Observatory of Economic Complexity
CBI	Centre for the Promotion of Imports from Developing Countries
HSDB	Hazardous Substances Data Bank
IARC	International Agency for Research on Cancer
pXRF	Portable X-Ray Fluorescence
PRISMA	Preferred Reporting Items for Systematic Reviews and Meta-Analyses
ICP-MS	Inductively Coupled Plasma-Mass Spectrometry
ICP-OES	Inductively Coupled Plasma-Optical Emission Spectroscopy
FSSAI	Food Safety and Standards Authority of India
GPC	Gel Permeation Chromatography
TLC	Thin-Layer Chromatography
LIBS	Laser-Induced Breakdown Spectroscopy
NIR	Near-Infrared
FT-IR	Fourier-Transform Infrared
FT-MIR	Fourier Transform Mid-Infrared
PCA	Principal Component Analysis
PLS-DA	Partial Least Squares Discriminant Analysis
XRD	X-Ray Powder Diffraction
FAAS	Flame Atomic Absorption Spectrometry
ML	Maximum Limits
EU	European Union
EC	European Commission
US	United States
CDC	Centres for Disease Control and Prevention
TNSRC	Tepi National Spices Research Centre
LMIC	Low–Middle-Income Countries

## Appendix A

**Table A1:** 2022–2023 Trade value and trade value growth of export of turmeric (Curcuma) from African countries in 2023 (Source: Modified from the [21]).

Country	Trade Value in 2022	Trade Value in 2023	Trade Value Growth (%)	% Total Turmeric Exported in 2023	% Total Turmeric Exported in 2022
Burkina Faso	208	248	0.192308	0.00	0.00
Cote d’Ivoire	632	11,744	17.58228	0.20	0.01
Cameroon	2747	1656	−0.39716	0.03	0.04
Democratic Republic of the Congo	16	44	1.75	0.00	0.00
Djibouti	568,864	106,439	−0.81289	1.82	8.97
Egypt	293,936	194,513	−0.33825	3.33	4.64
Ethiopia	2,698,590	3,331,579	0.234563	57.11	42.56
Ghana	4958	1283	−0.74123	0.02	0.08
Kenya	121,391	107,934	−0.11086	1.85	1.91
Morocco	67,660	11,005	−0.83735	0.19	1.07
Madagascar	952,484	992,241	0.04174	17.01	15.02
Mauritius	2347	462	−0.80315	0.01	0.04
Malawi	1	106	105	0.00	0.00
Nigeria	1,000,216	439,084	−0.56101	7.53	15.78
Togo	14,044	2239	−0.84057	0.04	0.22
Tunisia	82,399	8888	−0.89213	0.15	1.30
Tanzania	115,966	10,292	−0.91125	0.18	1.83
Uganda	56,409	35,302	−0.37418	0.61	0.89
South Africa	354,072	530,539	0.498393	9.09	5.58
Angola	0	14	1	0.00	0.00
Botswana	0	6	1	0.00	0.00
Comoros	0	1	1	0.00	0.00
Algeria	0	113	1	0.00	0.00
Senegal	0	25,762	1	0.44	0.00
Somalia	0	19,897	1	0.34	0.00
Zambia	0	2274	1	0.04	0.00
Benin	11	0	−1	0.00	0.00
Republic of the Congo	42	0	−1	0.00	0.00
Guinea	5	0	−1	0.00	0.00
Mali	86	0	−1	0.00	0.00
Mozambique	205	0	−1	0.00	0.00
Namibia	334	0	−1	0.00	0.01
Niger	65	0	−1	0.00	0.00
Rwanda	910	0	−1	0.00	0.01
South Sudan	300	0	−1	0.00	0.00
Sao Tome and Principe	38	0	−1	0.00	0.00
Eswatini	1101	0	−1	0.00	0.02
Zimbabwe	3	0	−1	0.00	0.00

**Table A2:** 2022–2023 Trade value and trade value growth of import of turmeric (Curcuma) in African countries in 2023 (Source: Modified from the [21]).

Country	Trade Value in 2022	Trade Value in 2023	Trade Value Growth (%)	% Total Turmeric Exported in 2023	% Total Turmeric Exported in 2022
Angola	11,814	42,562	260.2675	0.13	0.03
Burundi	296	295	−0.33784	0.00	0.00
Benin	9874	28,179	185.3859	0.09	0.02
Burkina Faso	3427	921	−73.1252	0.00	0.01
Botswana	18,419	17,674	−4.04474	0.06	0.05
Central African Republic	11,963	30,698	156.6079	0.10	0.03
Cote d’Ivoire	333,805	261,674	−21.6087	0.83	0.83
Cameroon	130,723	83,244	−36.3203	0.26	0.33
Democratic Republic of the Congo	18,376	17,043	−7.25403	0.05	0.05
Republic of the Congo	4591	6710	46.15552	0.02	0.01
Comoros	1187	4173	251.5586	0.01	0.00
Cape Verde	6020	10,077	67.39203	0.03	0.02
Djibouti	73,657	63,978	−13.1406	0.20	0.18
Algeria	3,702,310	2,588,220	−30.0918	8.19	9.25
Egypt	4,365,953	2,419,589	−44.5805	7.65	10.91
Eritrea	170	100	−41.1765	0.00	0.00
Ethiopia	69,397	197,474	184.557	0.62	0.17
Gabon	35,713	7521	−78.9404	0.02	0.09
Ghana	28,990	30,569	5.446706	0.10	0.07
Guinea	3737	7021	87.87798	0.02	0.01
Gambia	3755	36,915	883.0892	0.12	0.01
Equatorial Guinea	5305	18,764	253.7041	0.06	0.01
Kenya	256,417	276,676	7.900802	0.88	0.64
Liberia	6353	8374	31.81174	0.03	0.02
Libya	4,251,963	3,045,085	−28.384	9.63	10.63
Lesotho	2944	4349	47.72418	0.01	0.01
Morocco	15,183,390	12,571,001	−17.2056	39.76	37.95
Madagascar	8750	6702	−23.4057	0.02	0.02
Mali	9421	44,200	369.1646	0.14	0.02
Mozambique	20,053	49,928	148.9802	0.16	0.05
Mauritania	13,550	17,321	27.83026	0.05	0.03
Mauritius	501,962	553,807	10.32847	1.75	1.25
Malawi	1439	5050	250.9382	0.02	0.00
Namibia	19,979	31,933	59.83282	0.10	0.05
Niger	2423	8814	263.7639	0.03	0.01
Nigeria	244,982	96,810	−60.4828	0.31	0.61
Rwanda	5994	1606	−73.2065	0.01	0.01
Sudan	133,041	97,640	−26.6091	0.31	0.33
Senegal	267,747	137,439	−48.6683	0.43	0.67
Saint Helena	1034	10,411	906.8665	0.03	0.00
Sierra Leone	328	1808	451.2195	0.01	0.00
Somalia	26,291	31,727	20.67628	0.10	0.07
South Sudan	83,965	50,009	−40.4407	0.16	0.21
Sao Tome and Principe	90	100	11.11111	0.00	0.00
Eswatini	123,493	105,624	−14.4696	0.33	0.31
Seychelles	96,711	88,542	−8.44682	0.28	0.24
Togo	3284	4093	24.63459	0.01	0.01
Tunisia	3,513,144	2,933,842	−16.4896	9.28	8.78
Tanzania	7514	14,841	97.51131	0.05	0.02
Uganda	128,914	127,493	−1.10229	0.40	0.32
South Africa	6,063,566	5,095,890	−15.9589	16.12	15.16
Zambia	15,745	34,378	118.3423	0.11	0.04
Zimbabwe	172,441	290,945	68.72148	0.92	0.43
Guinea-Bissau	0	40	100	0.00	0.00
Chad	0	101	100	0.00	0.00

**Table A3:** A spectrum of reported turmeric powder adulterants and detection methods globally from 2004 to 2024.

Country (Sample Origin)	Method of Detection	Adulterant Investigated	References
***ADULTERANT: LEAD (CHROMATE)***			
India (Allahabad)	Laser-induced breakdown spectroscopic (LIBS) technique	Lead and Chromium	[89]
India	India’s Food Safety and Standards Authority (FSSAI) Physical and Chemical Methods	Lead chromate,	[124]
Bangladesh	Semi-structured interviews and informal observations (N = 152 heads), Inductively coupled plasma-mass spectrometry (ICP-MS), and Portable/Handheld X-ray fluorescence analysis (pXRF)	Lead and Chromium	[54]
Bangladesh	ICP-MS (LOD 0.001 µg/g Pb)	Lead	[54]
Thailand	Raman spectroscopy with PLSR	Lead	[90]
India	FSSAI: Physical and Chemical Methods (Do-at-home tests)	Lead chromate,	[125]
Russia (Moscow)	Electrothermal atomic absorption spectroscopy (AAS)	Lead and Chromium	[126]
Netherlands	FT-Raman spectroscopy and PLSR model	Lead chromate	[79]
India	Powder X-ray diffraction (PXRD) method (LOD: 0.5%)	Lead chromate	[91]
India	Diphenylcarbazide (DPC) colourimetric assay	Hexavalent chromium	[47]
India	pXRF and ICP-MS	Lead	[47]
Bangladesh, Pakistan	pXRF and ICP-MS	Lead	[47]
South Asia (India, Sri Lanka, and Nepal)	pXRF analyzer (XRF, Olympus Delta DCC-4000). LOD = 2 ug/g Pb	Lead	[10]
South Asia (India, Sri Lanka, and Nepal)	ICP-MS (LOD = 0.01 µg/g). For samples with Pb levels > LOD for pXRF.	Lead and Chromium	[10]
South Asia (Pakistan)	Inductively coupled plasma-optical emissions spectrometry (ICP-OES, LOD = 0.5 µg/g)	Lead and Chromium	[10]
South Asia (India, Pakistan, Sri Lanka, and Nepal)	The molar ratio of lead to chromium was calculated using the molar mass of lead (207.2 g/mol) and chromium (51.9961 g/mol). A molar ratio of lead to chromium close to 1:1 is suggestive of lead chromate.	Lead chromate (for samples with Pb levels >LOD)	[10]
Bangladesh	Multi-faceted pre-/post-intervention as follows: (i) disseminating findings from scientific studies via news media that identified turmeric as a source of lead poisoning; (ii) educating consumers and businesspeople about the risks of lead chromate in turmeric via public notices and face-to-face meetings, and (iii) collaborating with the Bangladesh Food Safety Authority to utilize a rapid lead detection technology to enforce policy disallowing turmeric adulteration.	Lead	[59]
Egypt	Multivariate chemometric models with those of artificial intelligent (AI) networks (AIN) to enhance the selectivity of spectral data for rapid assay, along with the PLS model, artificial neural network (ANN), and genetic algorithm (GA)	Lead chromate,	[94]
India	LIBS and multivariate technique	Lead and Chromium	[48]
USA	Desktop-based	Lead (Lead chromate)	[72]
***Adulterant: METANIL YELLOW***			
India	Two-dimensional high-performance thin-layer chromatography (2D-HPTLC) method	Metanil yellow	[127]
India	Preliminary colour test and thin-layer chromatography (TLC)	Metanil yellow	[128]
India	India’s Food Safety and Standards Authority (FSSAI)’s Physical and Chemical Methods	Metanil yellow dye	[124]
India	Ultraviolet-visible (UV-vis) spectrophotometer	Metanil yellow	[42]
India (Allahabad)	Preliminary colour test and TLC	Metanil yellow	[129]
USA	Fourier transform (FT)-Raman and Fourier-transform infrared spectroscopy (FT-IR) Spectroscopy	Metanil Yellow	[130]
India	Machine vision-based approach with PCA	Metanil yellow	[131]
India	TLC (Isopropyl alcohol as a diluent)	Metanil yellow,	[132]
India	NIR spectroscopy with PCA and partial least square regression (PLSR)	Metanil yellow powder	[133]
India	FT-MIR Spectroscopy/UV-Vis Spectroscopy/Electrical impedance spectroscopy (EIS) technique	Metanil Yellow	[134]
India	Gradient reverse-phase high-pressure liquid chromatographic (LOD: 0.37–2.48 µg/mL)	Metanil yellow	[31]
USA	Handheld near-infrared spectrometer and PCA-SIMCA Modelling	Metanil Yellow	[135]
USA (Lincoln)	Handheld NIR spectrometer and Benchtop NIR spectrometer (LOD and LOQ for the handheld and benchtop were 0.33 and 1.10%, respectively) using partial least squares regression models	Metanil yellow	[136]
India	FSSAI: Physical and Chemical Methods	Metanil yellow,	[137]
India	FSSAI: Physical and Chemical Methods (Do-at-home tests)	Metanil yellow,	[125]
India	Benzimidazole Based Bifunctional Sensor	Metanil yellow	[68]
India	Deep neural network and random forests-driven computer vision framework	Metanil yellow	[138]
Iran	Molecularly imprinted polymer dispersive solid-phase extraction and visible light spectrophotometry	Metanil yellow	[139]
Philippines	ATR-FTIR spectroscopy was used in tandem with one-class support vector machine (OCSVM)	Metanil Yellow	[140]
South Asia (India, Pakistan, Sri Lanka, and Nepal)	liquid chromatography-mass spectrometry (LC-MS) (LOD = 0.05 mg/kg)	Metanil yellow	[10]
Egypt/Saudi Arabia	Fluorescence Europium doped carbon dots	Metanil yellow	[141]
India	X-ray diffraction (XRD) and scanning electron microscopy (SEM)	Metanil yellow traces	[98]
India	FSSAI: Physical and Chemical Methods	Metanil yellow	[142]
Canada	1H-NMR spectroscopy, in conjunction with multivariate	Synthetic Metanil yellow	[143]
India	FSSAI: Physical and Chemical Methods	Metanil yellow	[144]
Egypt	Multivariate chemometric models with those of artificial intelligent (AI) networks (AIN) to enhance the selectivity of spectral data for rapid assay, along with the PLS model, artificial neural network (ANN), and genetic algorithm (GA)	Metanil yellow	[94]
India	FSSAI: Physical and Chemical Methods	Metanil yellow	[145]
India	Ultra-high-performance liquid chromatography with photodiode array detector-based analytical method	Metanil yellow	[97]
USA	Liquid chromatography-tandem mass spectrometry (LC-MS/MS)	Metanil yellow	[146]
India	Low-resolution handheld NIR spectroscopic platform assisted with chemometric algorithm (PCA-DA and Soft Independent Modelling of Class Analogy (SIMCA))	Metanil yellow powder	[147]
India	LIBS and multivariate technique	Metanil yellow	[48]
Iran	NIR spectroscopy associated with chemometric models (Soft Independent Modeling of Class Analogy (SIMCA) mode)	Metanil yellow	[148]
India	UV-VIS spectrophotometer	Metanil yellow	[42]
India (Allahabad)	Preliminary colour test and TLC	Metanil yellow	[129]
***Adulterant: OTHER CHEMICALS DYES***			
Spain	High-resolution Nuclear Magnetic Resonance (^1^H-NMR) and chemometric treatment [Partial Least Squares-Discriminant Analysis (PLS-DA)].	Sudan dye I, II, III, and IV	[49]
India	High Performance Thin Layer Chromatography—Mass Spectrometry (HPTLC–MS)	Sudan dyes	[149]
India (Allahabad)	Preliminary colour test and TLC	Sudan III	[129]
Egypt	Gel permeation chromatography (GPC) and HPLC with diode array detection (HPLC-DAD)	Sudan dyes	[150]
India	NIR spectroscopy and PCA	Sudan dye I	[151]
India (Allahabad)	Preliminary colour test and TLC	Sudan III	[129]
USA	FT-IR spectroscopy with partial least square regression (PLSR) model	Sudan Red G dye	[130]
USA	Raman imaging and FT-IR spectroscopy	Sudan Red	[152]
India	Benzimidazole Based Bifunctional Sensor	Sudan I and II	[68]
USA	Liquid chromatography-tandem mass spectrometry (LC-MS/MS)	Sudan I and Sudan Red G	[146]
India	Ultra-high-performance liquid chromatography with photodiode array detector-based analytical method	Sudan I	[97]
India	Low-resolution handheld NIR spectroscopic platform assisted with chemometric algorithm (PCA-DA and Soft Independent Modelling of Class Analogy (SIMCA))	Sudan dye-IV	[147]
Pakistan	HPLC with a variable wavelength detector (VWD)	Sudan dyes (I-IV)	[50]
Philippines	ATR-FTIR spectroscopy was used in tandem with one-class support vector machine (OCSVM)	Sudan I	[140]
India	Multiple Random Forest coupled with a computer vision technique	Sudan dye-I	[138]
Iran	NIR spectroscopy associated with chemometric models (Soft Independent Modeling of Class Analogy (SIMCA) mode)	Sudan Red	[148]
India	TLC (Isopropyl alcohol as a diluent)	Aniline dyes	[132]
India	FSSAI: Physical and Chemical Methods	Aniline dyes	[137]
India	FSSAI: Physical and Chemical Methods	Aniline dyes	[145]
India	FSSAI: Physical and Chemical Methods	Aniline dyes	[142]
Philippines	ATR-FTIR spectroscopy was used in tandem with one-class support vector machine (OCSVM)	Orange II	[140]
Egypt	Multivariate chemometric models with those of artificial intelligent (AI) networks (AIN) to enhance the selectivity of spectral data for rapid assay, along with the PLS model, artificial neural network (ANN), and genetic algorithm (GA)	Acid orange 7	[94]
***Adulterant: OTHER TUMERIC SPECIES***			
India	Randomly amplified polymorphic DNA (RAPD) Analysis, Polymerase chain reaction (PCR) amplification of the isolated DNA	*Curcuma zedoaria*	[153]
India	DNA-based method (molecular method)	Other *Curcuma* spp.	[154]
India	Molecular Sequence Characterized Amplified Region (SCAR) markers	*Curcuma zedoaria* and *C. malabarica*	[155]
Algeria	Microscopic analysis and Multivariate analysis (Principal component analysis, PCA)	spp	[156]
India	PCR amplification (DNA barcoding)	*C. zedoaria*	[157]
Indonesia	1H-NMR metabolite fingerprinting and multivariate analysis (combined with chemometrics of PCA and orthogonal projections to latent structures-discriminant analysis, OPLS-DA)	*Curcuma manga*	[158]
Indonesia	1H-NMR spectroscopy-based metabolite fingerprinting in combination with multivariate analysis (PLS-DA and OPLS-DA)	*Curcuma heyneana* and *C. manga*	[159]
India	Near-infrared (NIR) spectroscopy with Multivariate calibrations including the nearest neighbour (kNN) and support vector machine (SVM) as well as PCA	Other turmeric powders	[160]
Indonesia	Spectroscopy proton-nuclear magnetic resonance (^1^H-NMR) based metabolite fingerprinting and chemometrics (PCA and PLS-DA)	*Curcuma heyneana*	[158]
Indonesia	FT-IR spectroscopy with Attenuated Total Reflection (ATR) and Chemometrics	*Curcuma zedoaria* and *C. xanthorrhiza*	[161]
USA	FT-IR spectroscopy with partial least square regression (PLSR) model	*White turmeric* (*C. zedoaria*)	[130]
Spain (Barcelona)	Targeted liquid chromatography coupled to high-resolution mass spectrometry (LC-HRMS) with the characterization and classification using PCA and PLS-DA calculated on the Eigenvector Research Stand Alone Chemometric Software (SOLO)	Other *Curcuma* spp.	[162]
USA	Raman imaging and FT-IR spectroscopy	*White turmeric* powder	[152]
India	FT-NIR spectroscopy-based metabolic fingerprinting (and PCA and supervised PLS-DA)	Other *Curcuma* spp.	[163]
Sri Lanka	Machine learning-based fraud detection web application	Mixed/fake turmeric powder	[164]
Thailand	NIR and Raman spectroscopies with PLSR models	Variation in curcuminoid levels for authentication	[165]
Canada	1H-NMR spectroscopy, in conjunction with multivariate statistical analysis	*Curcuma mangga* and *C. caesia*, and Synthetic curcumin	[147]
India	Gas Chromatography coupled with Mass Spectrometry (GC-MS) technique	Plant-based adulterants	[166]
Taiwan	Loop-mediated isothermal amplification (LAMP)	Other *Turmeric* species	[167]
USA	Carbon-14 and HPLC analyses as complementary methods	Synthetic curcumin	[168]
India	ICP-MS; HPLC-PDA (photodiode array) and HPTLC-DS (densitometry) based on unique patterns (targeting: CIMP-1, i.e., (1E,4Z)-5-hydroxy-1-(4-hydroxy-3-methoxyphenyl) hexa-1,4-dien-3-one) with ESI-MS/MS (for confirmation)	Synthetic curcumin (presence of Boron (B) found as a qualitative indicator of SC (>250.0 mg/kg) and CLE (<2.0 mg/kg) by ICP-MS)	[169]
***Adulterant: SPENT TURMERIC***			
India	Vis-NIR spectroscopy and machine learning (PA)	Spent turmeric	[170]
Northern Ireland, UK	FT-IR spectroscopy coupled with chemometric analysis (PCA, OPLS-DA, and PLS-DA) and micro-FT-IR imaging	Spent turmeric	[8]
Adulterant: CHALK			
India	Terahertz spectroscopy	Chalk powder	[171]
India	India’s Food Safety and Standards Authority (FSSAI)’s Physical and Chemical Methods	Chalk powder	[124]
India	FSSAI: Physical and Chemical Methods	Chalk	[137]
India	FSSAI: Physical and Chemical Methods (Do-at-home tests)	Chalk (yellow-coloured)	[125]
India	FSSAI: Physical and Chemical Methods	Chalk powder	[172]
India	FSSAI: Physical and Chemical Methods	Chalk	[142]
India	FSSAI: Physical and Chemical Methods	Chalk	[144]
***Adulterant: STARCH***			
India	PCR amplification (DNA barcoding)	Starch (cassava, wheat, barley, and rye)	[157]
India	FSSAI: Physical and Chemical Methods	Starch (maize, wheat, rice)	[137]
India	FSSAI: Physical and Chemical Methods (Do-at-home tests)	Starch (corn),	[125]
Korea (Chuncheon)	Molecular markers using quantitative real-time PCR	Starch (*Z. mays*, corn)	[173]
Sri Lanka	Ultraviolet-visible-near infrared (UV-vis-NIR) multispectral imaging and multivariate statistical analysis	Starch (large rice particles)	[174]
Brazil	IR spectroscopy and multivariate analysis (PCA)	Starch	[175]
India	Visible-NIR spectroscopy with Machine Learning Methods (logistic regression (LR), K-nearest neighbour (KNN), and support vector machines (SVM))	Starch	[176]
India	Vis-NIR spectroscopy with PCA	Starch	[177]
India	Enhanced CNN in combination with deep learning (DL)	Starch (rice powder)	[178]
India	NIR spectroscopy with PCA	Starch	[179]
India	Colour images	Starch	[180]
South Asia (India, Pakistan, Sri Lanka, and Nepal)	American Spice Trade Association Method 8	Starch	[10]
Belgium	A robust set of qPCR methods (molecular method)	Starch (corn, *Zea mays*) and four other botanicals	[181]
Philippines	ATR-FTIR spectroscopy was used in tandem with one-class support vector machine (OCSVM)	Starch (corn)	[140]
Iran	Visible and short wavelengths of NIR hyperspectral imaging (Vis-SWNIR-HSI) combined with different chemometric techniques [multivariate curve resolution-alternating least squares (MCR-ALS) and mean-field independent component analysis (MF-ICA)], coupled with PCA (to find the pattern of authentic samples) and PLS-DA (for discrimination of the of adulterants)	Starch (corn flour, rice flour, wheat flour, and zedoary)	[182]
India	Improved convolutional neural network (CNN)	Wheat flour	[183]
Russia (Moscow)	Species-specific PCR	Wheat DNA	[126]
Mexico	Cavity Perturbation Technique (CPT)	Starch and	[128]
Northern Ireland, UK	FT-IR spectroscopy coupled with chemometric analysis (PCA, OPLS-DA, and PLS-DA) and micro-FT-IR imaging	Starch (rice flour and corn flour)	[8]
Canada	1H-NMR spectroscopy, in conjunction with multivariate	Starch (cassava)	[147]
India	FSSAI: Physical and Chemical Methods	Starch (maize, wheat, rice)	[145]
India	Low-resolution handheld NIR spectroscopic platform assisted with chemometric algorithm (PCA-DA and Soft Independent Modelling of Class Analogy (SIMCA))	Starch (corn powder)	[147]
China	Front-face synchronous fluorescence spectroscopy (FFSFS) and fluorescence titration coupled with partial least square (PLS) (LOD 5%)	Starch (maize flour)	[184]
Iran	NIR spectroscopy coupled with chemometrics (PCA and PLSR)	Starch (wheat flour and bread powder)	[185]
Russia	Fourier transform near-infrared (FT-NIR) spectroscopy with principal component analysis (PCA) and partial least square regression (PLSR)	Starch (corn)	[186]
***Adulterant: OTHER ARTIFICIAL COLOUR AND BULKING AGENTS***			
India	FSSAI: Physical and Chemical Methods	Coloured saw dust	[187]
India	FSSAI: Physical and Chemical Methods	Yellow lead salts	[137]
India	FSSAI: Physical and Chemical Methods	Yellow lead salts	[142]
India	FSSAI: Physical and Chemical Methods	Yellow lead salts	[145]
India	FSSAI: Physical and Chemical Methods	Yellow lead salt,	[144]
India	FSSAI: Physical and Chemical Methods	Yellow soapstone powder	[144]
India	FSSAI: Physical and Chemical Methods	Yellow soapstone	[172]
India	FSSAI: Physical and Chemical Methods (Do-at-home tests)	Sawdust	[125]
Sri Lanka	Multispectral imaging	Tartrazine (synthetic yellow azo dye, E number—E102)	[188]
India	Multispectral images on the smartphone	Tartrazine-coloured rice flour	[179]
Iran	Improved convolutional neural network (CNN) with MLP, Fuzzy, SVM, GBT, and EDT algorithms	Chickpea powder and chickpea powder mixed with food colouring	[189]
Mexico	Cavity Perturbation Technique (CPT)	Egg-yellow colour	[128]
India	FSSAI: Physical and Chemical Methods	Artificial colour	[187]
India	Antenna-based sensor	Artificial yellow colour	[190]
India	FSSAI: Physical and Chemical Methods	Artificial colour,	[144]
Iran	NIR spectroscopy coupled with chemometrics (PCA and PLSR)	Pistachio hull	[185]
Egypt/Algeria	HPLC, UV, FT-IR and ^1^H NMR and HPLC with Chemometric analysis (principal component analysis (PCA) and hierarchical clustering analysis (HCA))	Authenticity check	[191]
